# Identification of Novel Translational Urinary Biomarkers for Acetaminophen-Induced Acute Liver Injury Using Proteomic Profiling in Mice

**DOI:** 10.1371/journal.pone.0049524

**Published:** 2012-11-14

**Authors:** Rachel P. L. van Swelm, Coby M. M. Laarakkers, Ellen C. van der Kuur, Eva Morava-Kozicz, Ron A. Wevers, Kevin D. Augustijn, Daan J. Touw, Maro H. Sandel, Rosalinde Masereeuw, Frans G. M. Russel

**Affiliations:** 1 Department of Pharmacology and Toxicology, Radboud University Nijmegen Medical Centre, Nijmegen, The Netherlands; 2 Department of Laboratory Medicine, Radboud University Nijmegen Medical Centre, Nijmegen, The Netherlands; 3 Streekziekenhuis Koningin Beatrix Ziekenhuis, Winterswijk, The Netherlands; 4 Department of Pediatrics, Radboud University Nijmegen Medical Centre, Nijmegen, The Netherlands; 5 Department of Chemistry and Pharmacochemistry, Vrije Universiteit, Amsterdam, The Netherlands; 6 Apotheek Haagse Ziekenhuizen, The Netherlands; 7 Hagaziekenhuis, Den Haag, The Netherlands; UGent/VIB, Belgium

## Abstract

Drug-induced liver injury (DILI) is the leading cause of acute liver failure. Currently, no adequate predictive biomarkers for DILI are available. This study describes a translational approach using proteomic profiling for the identification of urinary proteins related to acute liver injury induced by acetaminophen (APAP). Mice were given a single intraperitoneal dose of APAP (0–350 mg/kg bw) followed by 24 h urine collection. Doses of ≥275 mg/kg bw APAP resulted in hepatic centrilobular necrosis and significantly elevated plasma alanine aminotransferase (ALT) values (p<0.0001). Proteomic profiling resulted in the identification of 12 differentially excreted proteins in urine of mice with acute liver injury (p<0.001), including superoxide dismutase 1 (SOD1), carbonic anhydrase 3 (CA3) and calmodulin (CaM), as novel biomarkers for APAP-induced liver injury. Urinary levels of SOD1 and CA3 increased with rising plasma ALT levels, but urinary CaM was already present in mice treated with high dose of APAP without elevated plasma ALT levels. Importantly, we showed in human urine after APAP intoxication the presence of SOD1 and CA3, whereas both proteins were absent in control urine samples. Urinary concentrations of CaM were significantly increased and correlated well with plasma APAP concentrations (r = 0.97; p<0.0001) in human APAP intoxicants, who did not present with elevated plasma ALT levels. In conclusion, using this urinary proteomics approach we demonstrate CA3, SOD1 and, most importantly, CaM as potential human biomarkers for APAP-induced liver injury.

## Introduction

Drug-induced liver injury (DILI) is the leading cause of acute liver failure and remains difficult to predict due to the lack of adequate biomarkers [1]. Monitoring of hepatic function in patients receiving drugs of risk is mainly based on measuring serum liver enzymes such as alanine aminotransferase (ALT) [2]. These enzymes are not accurately predictive for DILI, because they can be detected only after damage has been instigated [3]. In addition, some drugs can increase plasma liver enzymes without actually causing liver damage, such as diclofenac and methotrexate [4], [5]. Therefore, there is a need for biomarkers that can detect DILI at the onset and can be used as a tool during drug development and monitoring of patients [6]. Biomarkers predictive for DILI that can be detected in urine could be of great value to monitor patients on a regular basis in a non-invasive way. The urinary proteome mirrors the protein pool present in blood, and proteins related to pathologies, such as acute liver injury, can be detected in urine [7], [8]. Compared to blood, urine is well suited for proteomic profiling as it contains less high abundant proteins that can hamper biomarker detection [9]. Nevertheless, human sample collection for biomarker assessment is difficult, because the overall incidence of DILI is 10–15 cases in 100 000 patient years and the incidence for any particular drug can range from 1 case in 10.000 to 1.000.000 patient years [10].

Acetaminophen (APAP) is an interesting model compound for searching biomarkers related to acute DILI. APAP is metabolized to its reactive metabolite N-acetyl-*p*-benzoquinone imine (NAPQI), which is detoxified by conjugation to GSH. With high dosages of APAP, the GSH pool is depleted allowing NAPQI to bind to cellular macromolecules. Binding of NAPQI to mitochondrial proteins initiates the formation of reactive oxygen species and peroxynitrite. It has been demonstrated that oxidative stress leads to lipid peroxidation, mitochondrial dysfunction, disruption of calcium homeostasis and eventually necrotic cell death [11], [12]. Previous proteomics studies using rodent plasma and liver tissue showed marked changes in the expression levels of various proteins as a result of APAP-induced hepatotoxicity [13], [14], [15], including proteins involved in lipid/fatty acid metabolism, energy metabolism, oxidative stress, calcium homeostasis and inflammation.

The goal of this study was to identify proteins in human urine related to acute DILI. To this end, we implemented a translational approach to identify urinary biomarkers for human DILI. By first identifying proteins related to liver injury in urine of mice exposed to the drug of interest, and subsequently searching for the orthologous proteins in human urine, we aim to more efficiently use the limited availability of human urine samples for biomarker assessment. Here, we show carbonic anhydrase 3 (CA3), superoxide dismutase 1 (SOD1) and calmodulin (CaM) as potential urinary biomarkers for APAP-induced liver injury in both mouse and human.

## Materials and Methods

### Ethics statement

All experiments were approved by the local Animal Welfare Committee of the Radboud University Nijmegen (RU-DEC 2008-142 and RU-DEC 2009-101), in accordance with the guidelines of the Principles of Laboratory Animal Care (NIH publication 86-23, revised 1985).

Human sample collection was evaluated by the ethical committee of the Radboud University Nijmegen Medical Centre and the Hagaziekenhuis (Den Haag, the Netherlands) and they concluded that the performed research was not conducted under the regulations of the Act on Medical Research Involving Human Subjects, because sample collection included non-invasive sampling of urine and use of leftover plasma samples, taken for clinical analysis. Moreover, samples were collected anonymously and no clinically relevant or incriminating information were used. Written informed consent, therefore, was not compulsory; however, oral informed consent was obtained for all volunteers, patients and the parents of the underage patient with acetaminophen intoxication, which was not recorded to keep the procedure anonymous.

### Animal experiment

Male FVB mice (Charles River, Germany; 22–28 g bw) were housed under controlled conditions and randomly assigned to a single i.p. injection of vehicle (saline, n = 19)) or 100 (n = 6), 225 (n = 18), 275 (n = 33) or 350 (n = 6) mg/kg bw APAP (A500 Sigma-Aldrich Chemie B.V., Zwijndrecht, the Netherlands). As a negative control, mice (n = 6) were treated with 350 mg/kg bw 3-acetamidophenol (AMAP; A7205, Sigma-Aldrich). After injection, mice were placed individually in metabolic cages (Techniplast, Germany GmbH) to collect 24 h urine samples, with water and pulverized standard chow *ad libitum*. Protease inhibitors (Complete Mini, Roche Diagnostics, Almere, the Netherlands) were added to the urine, which was then centrifuged at 3000× *g* for 10 min at 4°C. Subsequently, blood plasma was collected in lithium-heparin tubes by eye extraction under isoflurane anesthesia and animals were sacrificed by cervical dislocation. Urine creatinine and plasma ALT levels were assessed by routine assays.

### Human sample collection

First, a control master pool was created consisting of 24 urine samples of both male and female volunteers between 18–65 years of age. Next, we were able to collect urine of a severe APAP intoxication, concerning a 5 year old girl of 12.5 kg bw that ingested approximately 12 tablets of 500 mg APAP. We received one urine sample collected upon hospital admission (urine sample 1) and one pooled urine sample composed of urine collected previous to, during, and after N-acetyl cysteine treatment (urine sample 2). Plasma liver enzymes were determined at hospital admission (plasma sample 1) and within 24 h after admission (plasma sample 2). Plasma liver enzyme values of both plasma samples were substantially increased. Enzyme concentrations in sample 1 and sample 2 were: ALT 8475 U/L and 9265 U/L (reference value <35), aspartate aminotransferase 16850 U/L and 18420 U/L (ref <40), lactate dehydrogenase 16010 u/L and 17730 U/L (ref 110–295) and gamma glutamyl transpeptidase 63 U/L and 60 U/L (ref <35), respectively.

In addition, plasma and urine samples were collected from 10 patients with suspected drug-induced acute liver injury that were admitted to the emergency room at Radboud University Nijmegen Medical Centre (Nijmegen, the Netherlands) and the Hagaziekenhuis (Den Haag, the Netherlands). The demographics of these patients are shown in Table 1.

**Table 1:** Demographics acute DILI patients.

Parameter	Reference value	APAP intoxicants	DILI 1	DILI 2
**Sex**			Female	Female
• **Female**		7		
• **Male**		1		
**Age**		39 (±17)	66	85
**Plasma ALT (U/L)**	<35	19 (±7)	217	269
**Plasma creatinine (µmol/L)**	60–120	54 (±18)	64	144
**Use of alcohol**			No	No
• **Yes**		1		
• **No**		7		
**Use of other drugs**			Yes	Yes
• **Yes**		3		
• **No**		5		
**Other drugs used**		DiazepamIbuprofenCoffeine	Amoxicillin and clavulanic acidOmeprazolAlprazolamZoldipemAlendronic acid	Co-trimoxazolPantoprazolLercanidipineDipyridamolAcetylsalicylic acidFurosemideMetoprolol

Mean values for the APAP intoxicants are represented as mean ± SD.

### Histology

Hematoxylin and eosin staining was performed on liver paraffin sections. Liver damage was evaluated by a qualified pathologist and scored blinded of 10 images taken from each liver section (10× magnification). For each image the degree of centrilobular necrosis was assessed by overlaying the images with a grid (Image J, 3700∧2 pixels) and counting the intersections in necrotic areas. Liver injury was reported as mean percentage of centrilobular necrosis. Kidney paraffin sections were stained using Periodic Acid Schiff staining.

### Urine protein profiling with MALDI-TOF MS

Urine samples were normalized according to creatinine values to reduce sample protein variation [16]. Based on the method of Fiedler *et al.*, urine samples were subsequently pretreated using affinity beads to isolate specific fractions of the urine proteome, before MALDI-TOF MS analysis [17]. We used weak cation exchange (WCX) Macro-Prep® carboxymethyl support beads (Bio-Rad Laboratories, Hercules, CA, USA) and Magnetic Beads based Hydrophobic Interaction Chromatography 8 beads (C8; Bruker Daltonics GmbH, Bremen, Germany), that bind positively charged proteins and hydrophobic proteins, respectively. Synthetic hepcidin-24 (Peptide International Inc., Louisville, KY, USA) was used as internal standard (IS) to enable comparison between samples. Of the prepared sample, 1 µl was applied to a MSP 96 polished steel MALDI target plate under nitrogen flow, followed by two times 0.5 µl of 5 mg/mL α-cyano-4-hydroxy-cinnamic acid in 50% ACN and 0.5% TFA. Mass-to-charge (m/z) spectra were generated using MALDI-TOF MS (Microflex LT with software flexControl Version 3.0, Bruker Daltonics) in positive, linear ion mode and 350 laser shots. Initial laser power; 50% for 1–20 kDa and 60% for 10–160 kDa measurements, Laser Attenuator; Offset 25% and Range 20%. Pulsed ion extraction was set to 250 ns. Samples prepared with the WCX support beads were measured in the 1–20 kDa mass range and those prepared with the C8 beads were measured in both the 1–20 kDa and 10–160 kDa mass range. Calibration was performed using protein calibration standard I for 1–20 kDa measurements and protein calibration standard II (both Bruker Daltonics) for 10–160 kDa measurements.

### Peptide and protein identification

Proteins were identified by using a MALDI linear ion trap mass spectrometer (vMALDI LTQ; Thermo Fisher Scientific) and LC-MS/MS (nLC LTQ FT Ultra MS; Thermo Fisher Scientific) as described elsewhere [18], [19]. Single C8 pretreated urine samples were used to identify specific protein masses smaller than 4 kDa directly with vMALDI LTQ. Proteins larger than 4 kDa were identified using 1D-gelelectrophoresis with a 15% SDS gel and silver-blue staining. Bands were excised and subjected to reduction, alkylation and trypsin digestion before being measured on the vMALDI-LTQ. For LC-MS/MS two pooled urine samples were used to identify differentially excreted proteins between control (n = 5) and APAP-induced liver injury (n = 5; plasma ALT>5000 U/L). Urine samples were in-solution digested, after reduction and alkylation. The digested samples were loaded on stagetips for desalting and concentrating, and eluted to a final volume of 20 µL, 8 µL of which was used for analysis. To avoid contamination with polymers, an extra strong cation exchange purification step was performed.

Database searches were performed using the Mus musculus RefSeq36 protein database supplemented with known contaminant proteins. For vMALDI LTQ the data search was performed using SEQUEST (v. 28 Bioworks™), for LC-MS/MS protein identifications were extracted from the data by means of the search program Mascot (v2.2; Matrix Science). The following modifications were allowed in the search: carbamidomethylation of cysteines (fixed), oxidation of methionine (variable) and acetylation of the N-terminus (variable). When appropriate, searches specified tryptic specificity, allowing a single missed cleavage site. Additional parameters for vMALDI LTQ were 1.4 Da precursor ion mass tolerance and 1 Da fragment ion mass tolerance. For LC-MS/MS precursor ion mass tolerance was set to 10 ppm, and fragment ion mass tolerance was set to 0.8 Da. Proteins identified using vMALDI LTQ were considered significant with a peptide probability >1^e^-002, and a protein probability >1^e^-003. Validation of proteins identified using LC-MS/MS was performed by an in-house developed script (PROTON) as described elsewhere [19].

### Immunoprecipitation

Urine samples were pretreated with C8 beads and incubated overnight with PBS and 0.1% Triton containing 2 mM CaCl_2_ and CaM antibody at 4°C. Subsequently, magnetic beads that bind IgG (Magnabind™ Protein G Magnetic beads, Thermo scientific, Rockford USA) were added and incubated for two hours at RT. After removal of the unbound fraction, proteins were eluted from the beads using 50% ACN and 0.5% TFA. The eluted fraction was measured using MALDI-TOF MS, as described. To correct for non-specific binding to the magnetic IgG beads, a urine sample was analyzed as described without CaM antibody.

### Western blot

Urine samples were normalized to creatinine (mouse samples) or protein concentration (human samples) before loading on a SDS gel. Antibodies against CA3 (1∶100), SOD1 (1∶2000) and CaM (1∶1000) were purchased from Abcam (Cambridge, UK). The following positive controls were used: recombinant human CA3 protein (Abcam), bovine SOD1 protein (Bruker Daltonics), and recombinant Xenopus laevis CaM [20]. Image J software (1.42q, National Institutes of Health, USA) was used to measure signal intensities on Western blot.

### ELISA assay

CaM concentration in human urine samples was determined using an ELISA assay (E90640Hu, Uscn Life Science, China) according to manufacturer's protocol. CaM concentrations were normalized to urine creatinine concentration. Samples were measured in duplicate.

### Statistical analysis

Statistics were performed using GraphPad Prism 5.02 (La Jolla, USA), unless indicated otherwise. A p-value of less than 0.05 was considered statistically significant. Data was compared between groups using one-way ANOVA with a post hoc multiple comparisons test. Spectra generated with MALDI-TOF MS were analyzed using flexAnalysis Version 3.0 and ClinProTools Version 2.2 software (both; Bruker Daltonics). Protein masses that differed significantly between the treatment groups were indicated using a Student's t-test or Wilcoxon rank test, depending on normal distribution. Relative peak intensities were calculated by dividing protein peak intensity by the peak intensity of the IS.

## Results

### Dose-dependent acute liver injury by APAP

**Figure 1:**
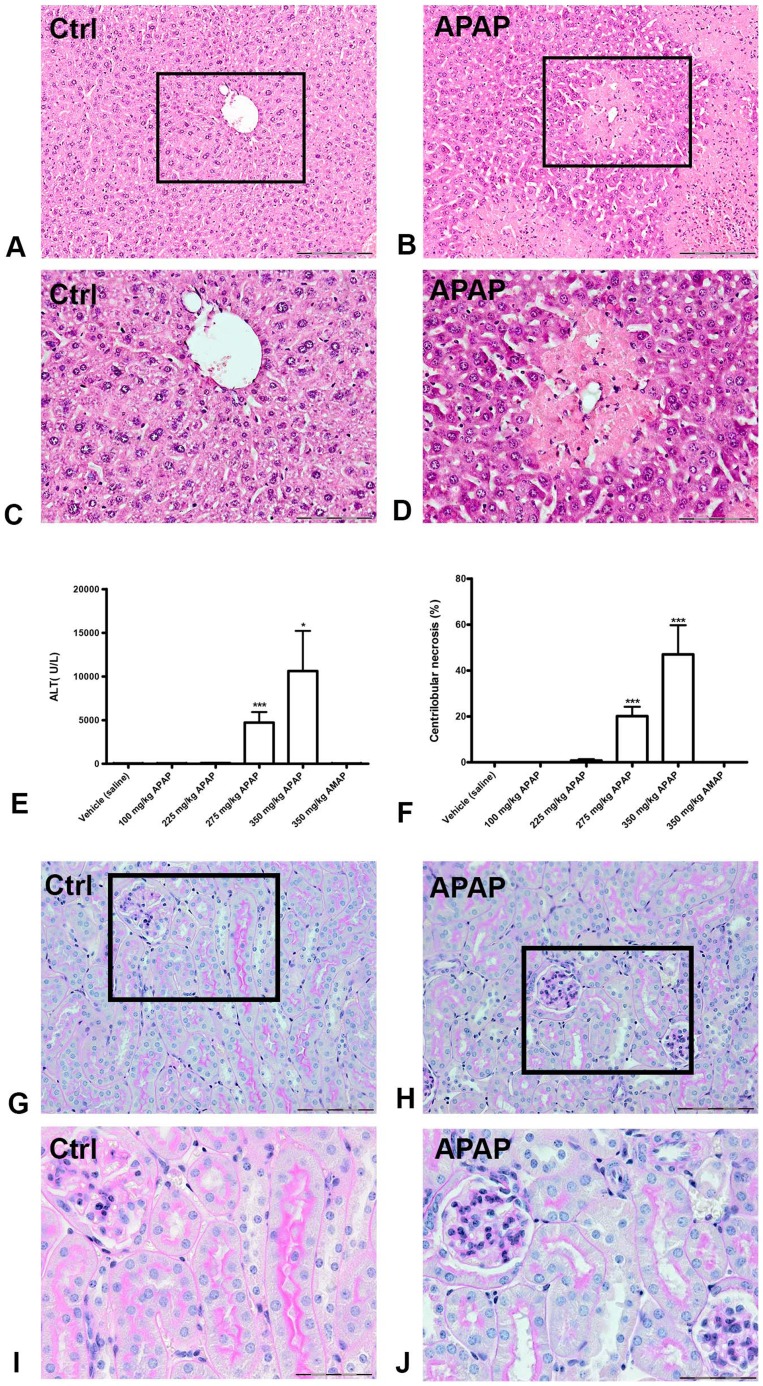
APAP-induced liver injury and kidney histology in mice. Hematoxylin and eosin staining of representative liver slides from a vehicle-treated mouse (A and C) and an APAP-treated mouse (B and D). Panels A and B show a 10× magnification, and a 20× magnification of the framed area is given in panels C and D, respectively. Centrilobular necrosis can be observed in liver slides after APAP treatment. Plasma ALT levels (E) and the percentage of centrilobular necrosis (F) increased significantly in mice receiving 275 and 350 mg/kg APAP. Periodic acid-Schiff staining of representative kidney slides from a vehicle-treated mouse (G and I) and an APAP-treated mouse (H and J) show no difference in histology. Panels G and H demonstrate a 20× magnification and a 40× magnification is given for the framed areas in panels I and J. The scalebar represents 200 µm in the slides with 10× magnification, 100 µm with 20× magnification and 50 µm with 40× magnification. ** *P*<0.01, *** *P*<0.001 compared to vehicle treated mice. ALT: alanine aminotransferase; AMAP: 3-acetamidophenol; APAP: acetaminophen.

Exposing mice to APAP resulted in dose-dependent hepatotoxicity, defined histologically as centrilobular necrosis (Figure 1A–D). The percentage of necrosis and the plasma ALT values were significantly increased after APAP administration compared to control and AMAP (Figure 1E and 1F). There was substantial interindividual variability in the toxic response to the highest doses of APAP reflected by the range in ALT values (40-29000 U/L) and percentage of necrosis (0–87%). Hence, ALT values and necrosis showed a strong intra-individual correlation. Renal tissue was analyzed histologically to rule out APAP-induced nephrotoxicity as a possible cause of altered urine proteome composition. We did not observe any histological changes that indicated kidney injury (Figure 1G–J).

### Presence of SOD1, CA3 and CaM in urine is related to APAP-induced liver injury in mice

**Figure 2:**
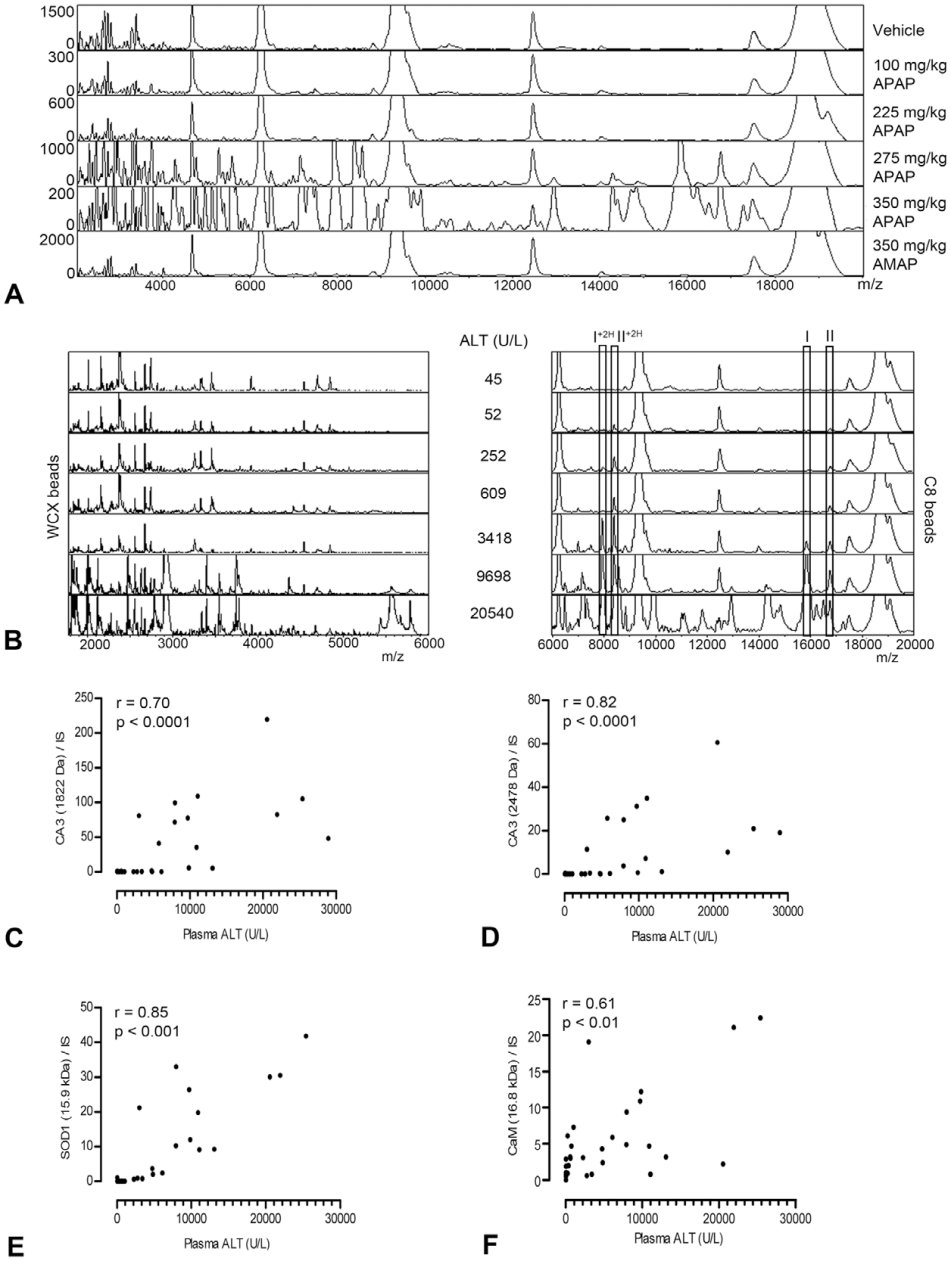
Urinary protein profiles of APAP-induced liver injury in mice. Representative urine protein profiles of *m/z* values versus peak intensity illustrate an APAP dose-related increase in urinary protein excretion (A). ALT-dependent increases in protein peaks were observed in urine samples pretreated with WCX beads or C8 beads (B). The protein masses of 15.9 kDa and 16.8 kDa are indicated by (I) and (II), respectively. Double charged forms are indicated by (+2H). The correlation between the relative peak intensity of two representative urinary CA3 fragments (C & D), SOD1 (E), and CaM (F) and plasma ALT was determined using the Spearman's rank correlation coefficient (r) in mice with APAP dose ≥275 mg/kg body weight. ALT: alanine aminotransferase; APAP: acetaminophen; CA3: carbonic anhydrase 3; CaM: calmodulin; SOD1: superoxide dismutase 1; WCX: weak cation exchange.

After urine profiling, an increased abundance in protein peaks was observed for mice treated with 275 and 350 mg/kg APAP compared to control and AMAP (Figure 2A). In total, 66 protein peaks in the WCX beads spectra, and 75 protein peaks in the C8 beads spectra were detected as significantly different between all APAP treatments and control. These proteins presented with increasing peak intensities in urine of mice with elevated plasma ALT values (Figure 2B). Most protein peaks were only detectable in urine of mice with relatively severe APAP-induced liver injury. However, two proteins of 15.9 and 16.8 kDa, later identified as SOD1 and CaM, respectively, were observed in the C8 beads spectra at low plasma ALT levels.

The eleven differentiating proteins that were identified using vMALDI LTQ are depicted in Table 2. LC-MS/MS analysis confirmed the presence of these proteins and additionally retrieved the identity of the 16.8 kDa protein, which was not found using vMALDI-LTQ (Table 3). Besides SOD1 and CaM, also peak intensities of fragments of CA3 correlated closely with plasma ALT values (Figure 2C–F), and therefore these 3 proteins were investigated further.

**Table 2:** Proteins identified with vMALDI-LTQ.

Protein	Protein Mass (Da)	P (pro)	Peptide sequence	[M+H]1+ (Da)
D-dopachrome tautomerase	13068.8	3.5e-007	R.FFPLEAVVQIGK.K	1335.7096
			K.FLTEELSLDQDR.I	1465.7169
			R.LCAATATILDKPEDR.V	1673.8527
			K.STEPCAHLLVSSIGVVGTAEQNR.T	2425.2140
Fatty acid binding protein 1 liver	14236.5	3.3e-007	K.AIGLPEDLIQK.G	1196.6885
			K.YQLQSQENFEPFMK.A	1788.8261
			K. SVTELN#GDTITNTMTLGDIVYK.R	2386.1694
PRED. Sim to superoxide dismutase 1	15974.8	9.7e-007	R.HVGDLGNVTAGK.N	1167.6117
			R.VISLSGEHSIIGR.T	1367.7641
			K.GDGPVQGTIHFEQK.A	1512.7441
Peroxiredoxin precursor 5	21883.5	1.2e-006	K.ATDLLLDDSLVSLFGNR.R	1848.9702
Glutathion-S-transferase π1	23594.1	7.8e-007	R.EAAQMDMVNDGVEDLR.G	1792.7840
			K.FEDGDLTLYQSNAILR.H	1854.9232
			K.ALPGHLKPFETLLSQN#QGGK.A	2136.1448
Glutathion-S-transferase α3	25344.3	1.5e-006	K.SHGQDYLVGNR.L	1245.5971
			R.ADIALVELLYHVEELPPGVVDN#FPLLK.A	3022.6023
Glutathion-S-transferase μ3	25685.0	2.9e-004	K.VTYVDFLAYDILDQ#YR.M	1994.9746
Glutathion-S-transferase μ1	25953.1	1.1e-006	R.YTMGDAPDFDR.S	1287.5310
			R.MLLEYTDSSYDEKR.Y	1749.8000
Carbonic anhydrase 3	29347.7	1.0e-007	R.VVFDDTYDR.S	1129.5160
			K.GEFQILLDALDK.I	1361.7311
			K.YAAELHLVHWNPK.Y	1577.8223
			R.EKGEFQILLDALDK.I	1618.8687
			K.HDPSLQPWSASYDPGSAK.T	1942.8930
			K.YN#TFGEALKQPDGIAVVGIFLK.I	2381.2751
			R.SLFSSAEN#EPPVPLVGNWRPPQPVK.G	2746.4199
Ketohexokinase	32719.5	3.4e-004	K.HLGFQSAVEALR.G	1327.7117
			K.VVHIEGR.N	910.4894
Regucalcin	33385.5	4.4e-007	R.WDTVSNQVQR.V	1232.6018
			R.VAVDAPVSSVALR.Q	1283.7318
			R.HQGSLYSLFPDHSVK.K	1714.8547
			R.HQGSLYSLFPDHSVKK.Y	1842.9497
			R.YFAGTMAEETAPAVLER.H	1855.8895

For each protein identified by vMALDI-LTQ the protein mass and the protein probability (P(pro)) are given. The peptide sequences by which the protein was identified are listed with their corresponding monoisotopic mass ([M+H]1+).

**Table 3:** Proteins identified with LC-MS/MS.

Protein	Reference	emPAIAPAP/C	PeptidesAPAP/C
D-dopachrome tautomerase*	gi|6753618|ref|NP_034157.1|	6.7	7
Fatty acid binding protein liver 1*	gi|8393343|ref|NP_059095.1|	214.4	6
Superoxide dismutase 1	gi|45597447|ref|NP_035564.1|	5.9	2
Peroxiredoxin precursor 5	gi|6755114|ref|NP_036151.1|	48.6	8
Glutathion-S-transferase π1*	gi|10092608|ref|NP_038569.1|	3.6	5
Glutathion-S-transferase α3*	gi|31981724|ref|NP_034486.2|	3.0	5
Glutathion-S-transferase μ1*	gi|6754084|ref|NP_034488.1|	10.5	11
Carbonic anhydrase 3*	gi|31982861|ref|NP_031632.2|	4.2	7
Ketohexokinase*	gi|31982229|ref|NP_032465.2|	0.8	4
Regucalcin*	gi|6677739|ref|NP_033086.1|	3.9	9
Calmodulin	gi|6753244|ref|NP_033920.1|	2.3	2

For each protein the ratio in protein abundance (emPAI) and number of unique peptides between mice with APAP-induced liver injury (APAP) and control (C) are given. Proteins completely absent in the control urine sample are indicated with *, in which case only the value for APAP is given.

**Figure 3:**
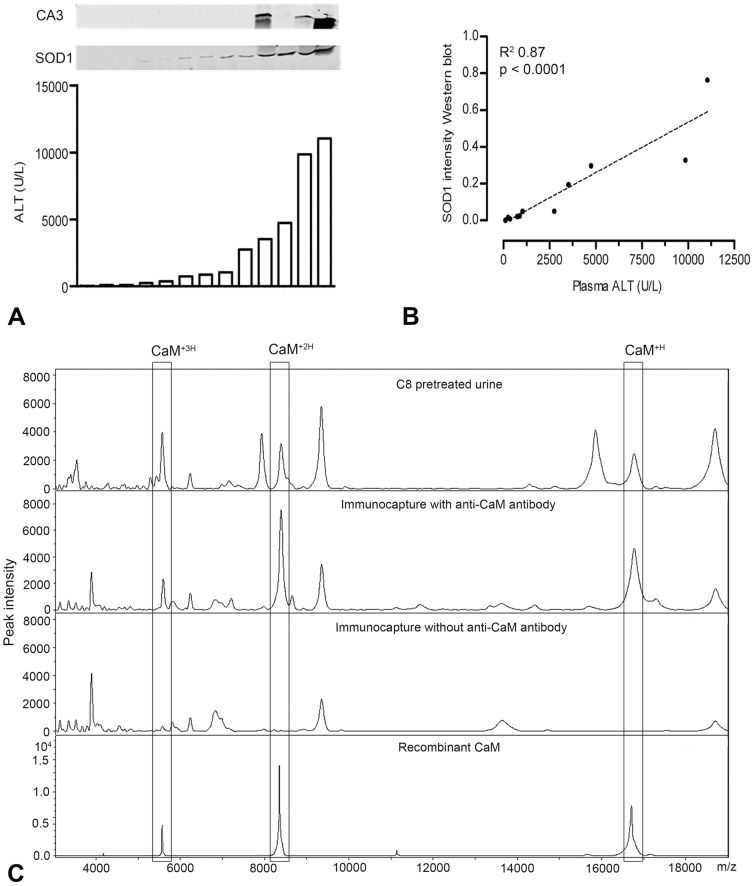
Identification of CA3, SOD1 and CaM in mouse urine. Western blots show the relation between urinary SOD1 and CA3, and plasma ALT levels in individual mice (n = 13; panel A), of which urinary SOD1 intensity on Western blot was analyzed by linear regression analysis (B). Immunoprecipitation demonstrated the specific protein profile of CaM, i.e. the mass peak for CaM at 16.8 kDa (CaM^+H^) and its double and triple charged form (CaM^+2H^ and CaM^+3H^), in mouse urine after APAP treatment (C). ALT: alanine aminotransferase; APAP: acetaminophen; CA3: carbonic anhydrase 3; CaM: calmodulin; SOD1: superoxide distmutase 1.

To confirm the presence of CA3 and SOD1 in urine by a specific antibody, we used Western blot analysis, as shown in Figure 3A. Whereas CA3 could be detected only in urine of mice with high plasma ALT (>3500 U/L) values, SOD1 was associated with minor elevations in plasma ALT (>100 U/L) and it gradually amplified with increasing plasma ALT values. After measuring the intensities of the SOD1 signal in the Western blot, linear regression analysis showed a significant correlation between urinary SOD1 and plasma ALT levels (Figure 3B). The third potential biomarker, CaM, was confirmed with an immunocapture assay, by which the 16.8 kDa peak was precipitated from C8 beads pretreated urine (Figure 3C), using a specific antibody against CaM.

### Urinary biomarkers identified in mice show potential for human acute DILI

**Figure 4:**
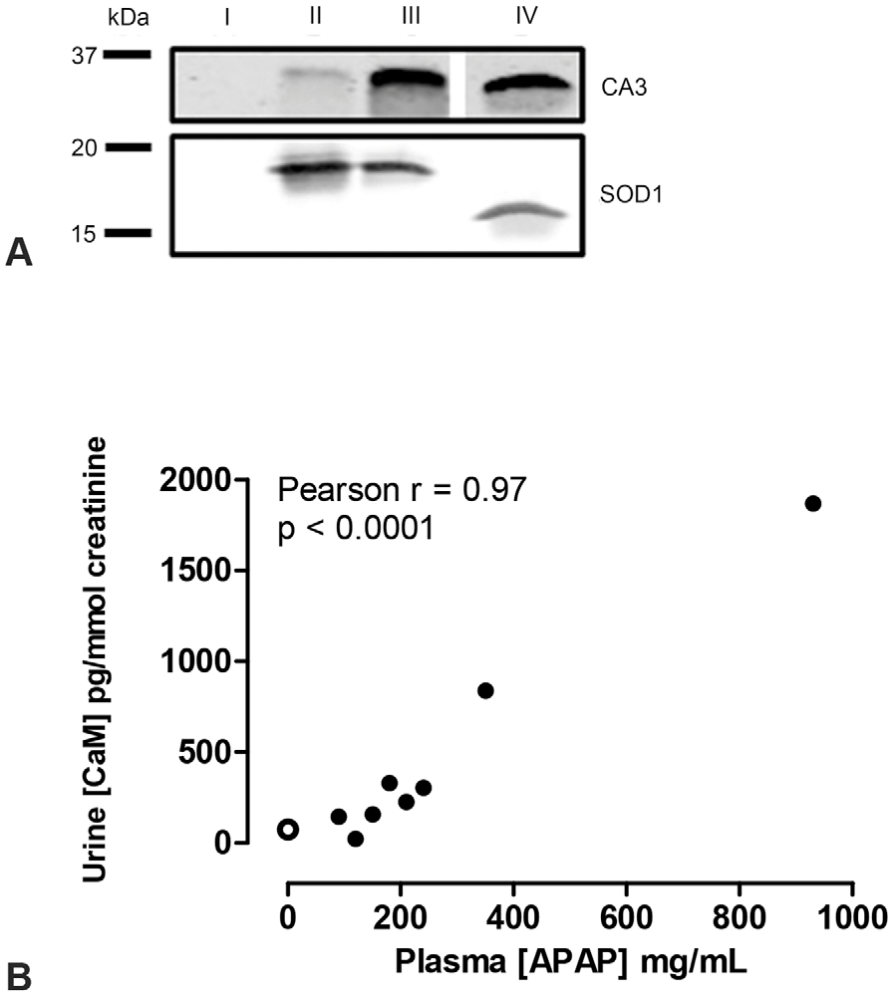
Detection of SOD1, CA3 and CaM in human urine samples. Presence of CA3 and SOD1 was assessed by Western blot in urine samples of masterpool control (I), severe APAP intoxication sample 1 (II) and 2 (III) and a positive control (IV) (panel A). Using an ELISA assay, the urinary concentration of CaM was correlated with plasma APAP concentrations using the Pearson correlation (r) test in patients with an APAP-intoxication, but without elevated plasma ALT values (B). The open data point represents the masterpool control urine sample. ALT: alanine aminotransferase; APAP acetaminophen; CA3: carbonic anhydrase 3; CaM: calmodulin; SOD1: superoxide dismutase 1.

To assess the biomarker potential of the proteins identified in relation to APAP-induced liver injury in mice, we analyzed urine samples of a patient with a severe APAP intoxication for the presence of CA3, SOD1 and CaM. Western blot analysis identified in both urine samples CA3 and SOD1, whereas both proteins were absent in the masterpool control sample (Figure 4A). Note that the positive control for SOD1 is of bovine origin and has a lower molecular weight than human SOD1. The concentration of urinary CaM, as determined by ELISA assay, was 75±15 pg/mmol creatinine (mean ± SD) in the masterpool control sample and increased to 150±5 pg/mmol creatinine in urine sample 1 and 3400±250 pg/mmol creatinine in urine sample 2.

Of all urinary proteins identified in mouse urine, CaM was the only protein found in urine of mice treated with a high dose of APAP that did not have elevated plasma ALT. Therefore, CaM could serve as an early biomarker for acute DILI. To further evaluate the potential of urinary CaM as novel biomarker for human acute DILI, we collected urine samples of patients that were admitted to the emergency room with suspected acute DILI. We collected urine of 8 patients with APAP intoxication and 2 patients with acute liver injury caused by other drugs, not including APAP (DILI 1 and DILI 2; Table 1). Although the patients with APAP intoxications did not show elevated plasma ALT levels, urinary CaM concentration was increased compared to the masterpool control sample and this increase correlated significantly with plasma APAP concentration (Figure 4B). Furthermore, urinary CaM concentration was increased in both patients with acute DILI not caused by APAP, to 140 pg/mmol creatinine in DILI 1 and 257 pg/mmol creatinine in DILI 2. These two patients, unlike the APAP intoxicants, did have elevated plasma ALT levels. To rule out acute kidney injury, plasma creatinine concentrations were measured, which were not increased in the APAP intoxicants and DILI 1, and only slightly elevated in DILI 2 (Table 2).

## Discussion

The present study was designed to identify novel biomarkers in urine for acute DILI by using APAP as model compound. Applying multiple proteomics techniques allowed us to identify twelve proteins related to APAP-induced liver injury. For the first time, we report the presence of CA3, SOD1 and CaM in urine to be related to APAP-induced liver injury, of which CaM had never been linked to liver injury before. Of these proteins, principally SOD1 and CaM closely associated with plasma ALT, as observed by proteomic profiling and antibody-based methods. CA3 fragments showed a good correlation with plasma ALT with proteomic profiling but this could not be confirmed using Western blotting with a specific antibody for the whole protein. However, CA3 as well as SOD1 and CaM were present in human urine samples after APAP intoxication, and are, therefore, proposed as potential urinary biomarkers for APAP-induced liver injury. Urinary CaM concentration was increased in human APAP intoxications and correlated well with plasma APAP concentration, whereas plasma ALT was not increased. This suggests that CaM might be an early marker compared to plasma ALT. Urinary CaM concentration was also elevated in two cases of human DILI caused by drugs other than APAP, indicating that CaM is not specific to APAP-induced liver injury, but rather to acute hepatocellular injury.

High doses of APAP caused liver damage as indicated by an increase in plasma ALT and centrilobular hepatic necrosis. Despite the use of inbred mice, our data indicate that the animals showed a differential response to APAP. This is most likely caused by a variation in glutathione stores in individual mice, since our mice were not fasted before APAP administration [21]. The variation in hepatotoxic response allowed us to correlate urinary protein levels to plasma ALT, a conventional biomarker of liver injury.

A major advantage of our experimental design was that we could profile proteins in urine collected in a controlled animal study. Urine samples from patients are difficult to profile in search for biomarkers, because they vary in many features. For example, nutritional status, disease condition, and/or use of other drugs may affect the urinary proteome. Using a translational approach, we were able to identify potential biomarkers for APAP-induced liver injury in mice and confirm the presence of these proteins in human urine samples after APAP intoxication and DILI caused by other drugs.

In mice, urine was collected during 24 h after APAP administration, and plasma and liver tissue samples at 24 h after exposure. We measured urine at one time point after APAP administration, but still observed a strong association between plasma ALT values and both SOD1 and CaM levels in urine samples. Yet, we could not assess if these potential biomarkers are excreted in urine early after the onset of injury. Nevertheless, SOD1 has previously been reported to appear in rat urine as early as 12 h after treatment with CCl_4_, another known hepatotoxic chemical [22]. A disadvantage of urine collection during 24 h could be that potentially interesting proteins are difficult to detect because of dilution, particularly those excreted shortly after the onset of injury. In addition, some proteins may be unstable in urine and only fragments rather than intact proteins can be detected. This has likely occurred for CA3 in the present study.

Obviously, the kidney has a major influence on urine content and approximately 70% of the proteins in urine originate from this organ [23]. Since most proteins identified in this study are not liver-specific, we investigated whether potential kidney injury by APAP could have been a confounding factor. No signs of kidney injury were observed after APAP treatment as determined by histology and the absence of kidney injury markers (kidney injury molecule-1 and neutrophil gelatinase associated lipocalin; data not shown). We, therefore, assume that the proteins found in urine after APAP-induced liver injury were not the result of kidney injury, but were released from liver into blood and subsequently excreted by the kidney. Most of the proteins identified in this study were only found in mice with high plasma ALT values and do not seem to be suitable as biomarker. Urinary CA3 and SOD1 showed a good correlation with plasma ALT and probably are also leakage markers of injured hepatocytes. The advantage over plasma ALT is that these markers can be measured in patients non-invasively. CaM proved to be the most promising biomarker, because the protein was found in urine of mice treated with a high dose of APAP that did not show elevated plasma ALT levels. This was also observed in urine samples of human APAP intoxicants. Although plasma ALT levels were not increased in these patients, plasma APAP concentrations were high enough that liver injury was a concern as indicated by the Rumack-Matthew normogram [24]. These data indicate that CaM has potential as predictive biomarker for acute DILI and that a mechanism of hepatocyte release other than leakage may be involved.

Most of the proteins that we detected in urine are involved in intracellular processes related to APAP-induced liver injury (Table 1 and 2) [25], [26], [27], [28]. These process are not specific to APAP and, accordingly, the biomarkers identified in this study are most likely not specific to APAP, but rather to acute hepatocellular injury. In line with this, urinary CaM concentration was also increased in human cases of DILI not caused by APAP. Since oxidative stress, mitochondrial damage and disrupted calcium homeostasis play an important role in APAP-mediated hepatotoxicity, it is not exceptional that we identified SOD1 and CaM as proteins with biomarker potential. The involvement of superoxide dismutases in APAP-induced liver injury has previously been demonstrated by the increased toxicity of APAP in mice with reduced activity of SOD2 [29], [30]. The exact role of SOD1 in APAP-mediated hepatotoxicity remains controversial as both protective and damaging effects have been reported, but SOD1 nitration and reduction in SOD1 activity appear to be involved [31]. A role for CaM in APAP-induced liver injury has not been clearly described; however, CaM does play a key role in maintaining intracellular calcium balance. Binding of NAPQI to mitochondrial proteins can cause mitochondrial permeability transition, after which mitochondrial Ca^2+^ is released into the cytosol [32]. The cytosolic Ca^2+^ concentration is tightly regulated and any excess Ca^2+^ will be effluxed via the plasma membrane Ca^2+^ ATPase transporter (PMCA), using CaM as ultimate cofactor [33]. However, the peroxynitrite formed during APAP-induced oxidative stress can oxidize specific methionine positions of CaM, after which CaM is no longer able to activate PMCA, which results in reduced excretion of cytosolic Ca^2+^ [34]. Previous studies showed decreased activity of PMCA during APAP-induced liver injury [35]. With sustained high cytosolic Ca^2+^ concentrations, Ca^2+^ will be translocated to the nucleus by CaM, where it will cause DNA fragmentation and ultimately lead to cell death [36]. CaM is thus involved in the initiating events of APAP-induced liver injury and may, therefore, be a potential early biomarker.

Based on these data, the next step would be to investigate the dynamics of the suggested biomarkers on hepatic regulation and excretion in urine with DILI, by including multiple time points of measurement after drug administration. This will also allow better comparison of the urinary proteins as non-invasive biomarkers with the conventional plasma ALT measurements, including the predictive value for DILI. Continuous urine sample collection of patients with APAP-induced liver injury and DILI caused by other drugs is needed to assess further the suitability of the biomarkers suggested for acute DILI in general.

In summary, using a translational approach we identified CA3, SOD1 and CaM as novel urinary biomarkers in relation to APAP-induced liver injury in both mouse and human urine samples. These results allow further clinical validation to assess their applicability as non-invasive biomarkers for acute DILI.
